# Factors Associated with Cardiorespiratory Fitness in a Swiss Working Population

**DOI:** 10.1155/2019/5317961

**Published:** 2019-07-02

**Authors:** Sara Kind, Stefanie Brighenti-Zogg, Jonas Mundwiler, Ulla Schüpbach, Jörg D. Leuppi, David Miedinger, Thomas Dieterle

**Affiliations:** ^1^University Clinic of Medicine, Cantonal Hospital Baselland, Liestal, CH, Switzerland; ^2^University Department of Radio-Oncology, lnselspital, Bern University Hospital, Bern, CH, Switzerland; ^3^Faculty of Medicine, University of Basel, Basel, CH, Switzerland

## Abstract

**Background:**

Good cardiorespiratory fitness (high VO_2max_) has beneficial effects on morbidity and mortality. Therefore, a tool to estimate VO_2max_ in daily clinical practice is of great value for preventing chronic diseases in healthy adults. This study aimed at exploring the cardiometabolic profile in a representative Swiss working population. Based on these insights, a regression model was derived revealing factors associated with VO_2max_.

**Methods:**

Cross-sectional data of 337 healthy and full-time employed adults recruited in the Basel region, Switzerland, were collected. Anthropometric measurements to compute body mass index (BMI) and waist circumference (WC) were performed. A 20-meter shuttle run test was conducted to determine individual VO_2max_. Heart rate (HR) was measured at rest, during maximal exertion, and two minutes after exercise. Systolic (SBP) and diastolic blood pressure (DBP) were assessed at rest and after exercise. A multiple linear regression model was built to identify a set of nonexercise predictor variables of VO_2max_.

**Results:**

Complete data of 303 individuals (63% male) aged 18 to 61 years (mean 33 ± 12 years) were considered for analysis. The regression model (adjusted R^2^ = 0.647, SE = 5.3) identified sex (*β* = -0.699, p < 0.001), WC (*β* = -0.403, p < 0.001), difference of maximal to resting HR (*β* = 0.234, p < 0.001), smoking (*β* = -0.171, p < 0.001), and age (*β* = -0.131, p < 0.01) as the most important factors associated with VO_2max_, while BMI, SBP, and DBP did not contribute to the regression model.

**Conclusions:**

This study introduced a simple model to evaluate VO_2max_ based on nonexercise parameters as part of daily clinical routine without needing a time-consuming, cost-intense, and physically demanding direct assessment of VO_2max_. Knowledge about VO_2max_ may help identifying individuals at increased cardiovascular risk and may provide the basis for health counselling and tailoring preventive measures.

## 1. Introduction

Physical inactivity is rising on a global scale, thus yielding dramatic consequences for the general health of the population and representing a huge burden to the healthcare systems [1]. According to the World Health Organization (WHO), inactive individuals have higher levels of body fat and are at higher risk for cardiovascular (CV) disease compared to regularly active persons [1]. Physical inactivity is estimated to be the fourth leading risk factor for mortality worldwide [1]. Nevertheless, previous studies indicated that cardiorespiratory fitness measured as maximal oxygen uptake (VO_2max_) is more closely correlated with CV risk factors than physical activity [2]. VO_2max_, defined as the maximum rate at which oxygen can be utilized by the body during maximal exertion, is usually given in ml of consumed oxygen per kg of body weight per min and values range from <20 ml/kg/min in inactive adults to 70-94 ml/kg/min in athletes [3]. Factors affecting VO_2max_ include oxygen diffusion capacity of the lungs, cardiac output, oxygen transport capacity of the blood, capillary density of the muscles, and muscular mitochondria mass [3]. VO_2max_ further depends on sex, age, genetics, body fat, medical conditions, and smoking [4–6]. Obesity was also found to correlate with lower maximal oxygen uptake and poorer cardiorespiratory fitness [7]. Longitudinal studies indicated that a low VO_2max_ is an independent and strong predictor of various diseases, such as stroke, hypertension, and metabolic syndrome [8, 9]. On the other hand, VO_2max_ values above 30 ml/kg/min correlate with a reduced relative risk of mortality [10].

The gold standard for measuring VO_2max_ is spiroergometry—a direct measurement of oxygen uptake during maximal exertion, usually by using a cycle ergometer or treadmill [11]. However, spiroergometry is time-consuming, requiring special equipment and skilled personnel [12]. In addition, the availability of appropriate infrastructure is limited, particularly in primary care settings. For this reason, alternative methods have been developed to estimate VO_2max_ on a large scale within the population [12]. These methods include submaximal exercise testing as well as mathematical models not requiring exercise at all [13–15]. However, most of the prediction models were developed for specific patient groups and are not cross-validated in larger populations.

Therefore, the present study aimed at exploring parameters reflecting metabolic and CV profile in a representative Swiss working population in order to identify a set of nonexercise variables associated with VO_2max_. The goal was to develop a simple model for daily clinical routine not requiring time-consuming, cost-intense, and physically demanding direct assessment of VO_2max_. Knowledge about VO_2max_ may help identifying individuals at increased CV risk and may provide the basis for health counselling and tailoring preventive measures. Early detection of increased CV risk might have a great impact on risk reduction, well-being, and work productivity in the general population.

## 2. Materials and Methods

### 2.1. Study Design

This cross-sectional analysis is based on data from a previous study by Brighenti-Zogg et al. [16], which enrolled healthy and full-time employed (≥80% full-time equivalent) individuals aged 18 to 65 years from various small and medium sized companies of the Basel region, Switzerland. Details on study design and recruitment are published elsewhere [16]. Exclusion criteria were missing informed consent, insufficient knowledge of the German language, movement restrictions, and diseases and accidents within the last three months that affected productivity at the workplace. The study was approved by the local ethics committee for northwest and central Switzerland (EKBB: 260/12) on December 21, 2012.

### 2.2. Study Procedures and Measurements

After providing written informed consent, participants underwent measurements of body height, weight, and WC during the study visit. Body weight was performed on volunteers in light clothing without shoes by a medical scale (model Seca 877, load capacity: 200 kg, Seca AG, Reinach, Switzerland) with an accuracy of 0.1 kg. Body height was assessed without shoes by a medical measuring stick (model Seca 217, measurement range: 20 to 205 cm, Seca AG, Reinach, Switzerland) to the nearest mm. The measurement of WC was determined midway between the lowest rib and the iliac crest using a medical measuring tape (model Prym, length: 150 cm, Germany) with a precision of 0.1 cm. Abdominal obesity was defined as >94 cm in men and >80 cm in women [17]. Measurements of height and weight were used to calculate BMI (BMI = weight / height^2^). Participants with a BMI of ≥30 kg/m^2^ were classified as obese [18]. Furthermore, personal and job-related factors were recorded by a generic questionnaire including age, sex, nationality, smoking status, profession, daily working hours, medication, current illnesses, and accidents. Then, participants had to perform a 20-meter shuttle run in order to measure VO_2max_ [19]. They were briefed to run forth and back between two lines with a distance of 20 meters according to audio signals. Starting speed was 8.5 km/h and every minute speed was increased by 0.5 km/h. Once the participant could no longer keep the pace (>3 meter away from the 20-meter line), the test was terminated. The number of reached stages and shuttles was used to estimate VO_2max_ [12]. VO_2max_ values were subdivided based on percentiles in order to classify participants into three fitness level groups (i) <25th percentile (<P25), (ii) 25th-75th percentile (P25-75), and (iii) >75th percentile (>P75). Heart rate (HR) was recorded before (resting HR), during (maximal HR), and two minutes after the exercise test (recovery HR) using a wrist-worn polar watch (model Polar RS300X sd, Polar Electro Oy, Kempele, Finland). The difference of maximal to resting HR, respectively, of maximal to recovery HR was calculated. In addition, age-predicted maximal HR was determined using the validated formula ‘208 – (0.7 x age)' by Tanaka et al. [20], which has been described as more accurate than the most common formula ‘220 – age' [21]. Systolic (SBP) and diastolic blood pressure (DBP) were measured in a standardized way according to the 2013 European Society of Hypertension (ESH) guidelines [22] using an automated BP device for use at the upper arm (Omron M6W, Omron Healthcare Co., Kyoto, Japan). One measurement each was taken at rest and during recovery (two minutes after the test). The double product was calculated as HR multiplied by SBP at rest. Following the study visit, daily physical activity was recorded with the Sense Wear Mini armband (BodyMedia Inc., Pittsburgh, PA, USA) on seven consecutive days (23 hours per day). As recommended by the WHO, participants were classified into groups with sufficient and insufficient activity levels based on the cut-off ≥30 minutes of moderate-to-vigorous physical activity (MVPA) per day [1].

### 2.3. Statistical Analysis

Statistical analysis was performed using SPSS Statistics (Version 22.0, IBM, Switzerland). Data are presented as mean and standard deviation (SD). Normal distribution of data was evaluated with the Shapiro-Wilk test. Differences between fitness level groups were analysed using One-Way Analysis of Variance (ANOVA) or Kruskal-Wallis test, if appropriate. Categorical data were analysed using the Chi-Square test. Bivariate correlations between measured and estimated values of VO_2max_ as well as maximal HR were calculated using Spearman's rho (one-sided). In order to identify the most important factors associated with VO_2max_, a forward-stepwise multiple linear regression analysis was performed including age, sex, smoking status, metabolic parameters (BMI, WC), vital signs (HR, SBP, DBP, double product), and physical activity level (MVPA) as independent variables based on previous research. Validity of the regression model was proved by checking essential assumptions (i.e., multicollinearity of independent variables, homoscedasticity, independence, and normality of errors). Statistical significance was set at the 5%-level.

## 3. Results

Complete datasets were available from 303 participants. Two-thirds (n = 190, 62.7%) were male and mean age was 33.5 ± 12.0 years, ranging from 18 to 61 years. Most of the participants (77.9%) were younger than 45 years. The majority were Swiss (72.9%), 23.1% European, and 4.0% other nationalities. Further characteristics are summarized in Table 1.

Mean VO_2max_ was significantly higher in men compared to women (p < 0.001). The fittest group showed a mean of 52.7 ± 3.7 ml/kg/min, the middle group a mean of 40.1 ± 4.3 ml/kg/min, and the unfit group a mean of 27.8 ± 3.1 ml/kg/min. Sex differences were evident across all fitness level groups (p < 0.05). Subjects in the fittest group (27.6 ± 8.4 years) were significantly younger than those in the middle (33.8 ± 12.0 years) and unfit group (38.1 ± 14.4 years) (p < 0.001). Mean BMI was significantly higher in men than in women (p < 0.001). While 6.9% of the participants were obese, 33.8% presented with abdominal obesity with a significant difference between men and women (p < 0.001). Individuals in the fittest group showed a trend towards lower BMI and WC than those in the middle and unfit group.

**Figure 1:**
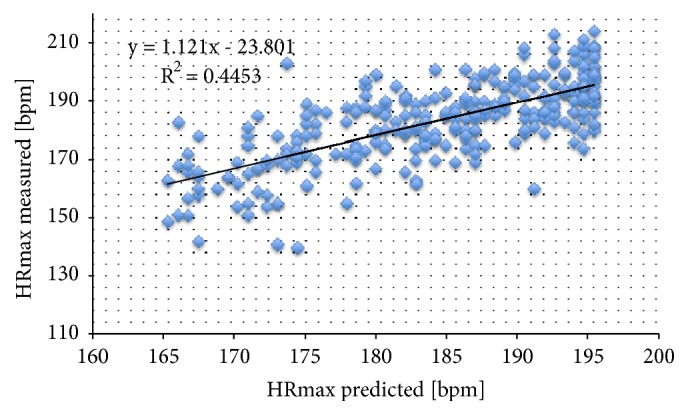
Scatterplot illustrating the correlation between HR_max_ measured during the 20-meter shuttle run test (Y-axis) and HR_max_ predicted using the formula of Tanaka et al. [20] (x-axis). HR_max_: maximal heart rate.

HR at rest was significantly higher in women compared to men (p < 0.05), respectively, in the unfit (78 ± 13 bpm) compared to the middle (71 ± 12 bpm) and fittest group (67 ± 13 bpm) (p < 0.001). In contrast, higher HR was observed in men at maximal exertion (p < 0.001) and during recovery (p < 0.01). Maximal HR was highest in the fittest group (191 ± 10 bpm) and lowest in the unfit group (174 ± 17 bpm) (p < 0.001). Mean age-predicted maximal HR (185 ± 9 bpm) [23] and measured maximal HR (183 ± 15 bpm) were strongly correlated (r = 0.699, p < 0.001), which is illustrated in Figure 1. Resting SBP was significantly higher in men than in women (p < 0.001), respectively, in the fittest compared to the unfit group (139 ± 12 vs. 129 ± 14 mmHg, p < 0.001). However, when stratified for sex, SBP did no longer differ between fitness level groups. SBP during recovery was also higher in the fittest (170 ± 16.7 mmHg) compared to the middle (164 ± 18.7 mmHg) and unfit group (156 ± 18.6 mmHg) (p < 0.01). DBP at rest and during recovery did not differ between the three fitness level groups. The double product was lower in the fittest compared to the unfit group, although not statistically significant.

**Figure 2:**
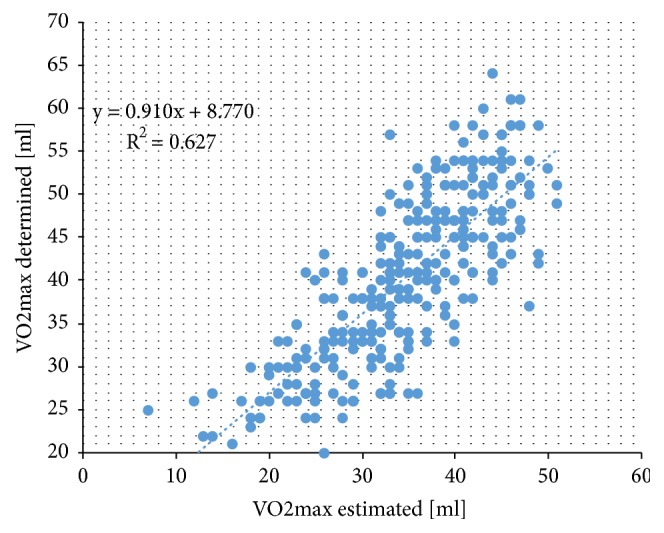
Scatterplot depicting the correlation between VO_2max_ determined by the 20-meter shuttle run test [12] (y-axis) and VO_2max_ estimated using the developed regression equation (x-axis). VO_2max_: maximal oxygen uptake.

Table 2 presents the results of the forward-stepwise multiple linear regression analysis with VO_2max_ as dependent variable. The overall fit of the regression model was high explaining 64.7% of variance (standard error (SE) = 5.3) in VO_2max_. In decreasing order, sex, WC, difference of maximal to resting HR, smoking, and age were the most important factors associated with VO_2max_. In contrast, BMI, SBP, DBP, difference of maximal to recovery HR, double product, MVPA, and daily working hours did not contribute significantly to the regression model and were therefore excluded. The strong and highly significant correlation (r = 0.805, p < 0.001) between VO_2max_ determined by the 20-meter shuttle run test and VO_2max_ estimated using the developed regression equation is depicted in Figure 2.

Based on these results, the following regression equation for estimating VO_2max_ was derived:

VO_2max_ [ml/kg/min] = 77.947 – (13.374 x sex; men = 1, women = 2) – (0.338 x waist circumference) + (0.121 x HR_max_-to-HR_rest_) – (3.938 x smoking; never smoker = 1, current smoker = 3) – (0.098 x age).

**Example 1 .**

A 20-year-old male nonsmoker with a normal WC of 80 cm and a low resting HR of 50 bpm would have the following VO_2max_, when calculating maximal HR using the formula of Tanaka et al. (208 – (0.7 x 20) = 194):

77.947 – (13.374 x 1) – (0.338 x 80) + (0.121 x 144) – (3.938 x 1) – (0.098 x 20) = 49.1 ml/kg/min

**Example 2 .**

A 50-year-old male smoker with a high-risk WC of 100 cm and a high resting HR of 80 bpm would have the following VO_2max_, when calculating maximal HR using the formula of Tanaka et al. (208 – (0.7 x 50) = 173):

77.947 – (13.374 x 1) – (0.338 x 100) + (0.121 x 93) – (3.938 x 3) – (0.098 x 50) = 25.3 ml/kg/min

While Example 1 representing a young and healthy male individual achieved a VO_2max_ close to the mean of the fittest group, VO_2max_ of Example 2 with a high-risk metabolic and CV profile was below the mean of the unfit group.

## 4. Discussion

The key finding of this cross-sectional analysis including data from 303 healthy Swiss employees was that participants in the fittest group were younger, had a lower BMI and WC, a higher difference of maximal to resting HR, a lower double product (HR x SBP), and were more physically active. Multiple linear regression analysis identified sex, WC, difference of maximal to resting HR, smoking, and age as the most important factors associated with cardiorespiratory fitness (VO_2max_), while SBP and DBP did not correlate with VO_2max_.

### 4.1. Metabolic and Cardiovascular Profile

BMI defined obesity was found in 9% of male and 4% of female participants. These rates are lower than those reported in the Swiss menuCH survey in 2015 [24]. The difference may be explained by the fact that our study included relatively young and healthy individuals. However, based on WC, one-third of the participants presented with abdominal obesity. WC provides a more reliable measure for obesity than BMI, as it is not confounded by muscle mass [25]. Moreover, it is more accurate in predicting morbidity and mortality by reflecting fat distribution and given the fact that intra-abdominal fat is the most metabolically active, CV risk [23, 26]. Despite this advantage, the measurement of WC may be challenging [27].

Resting HR was higher in women compared to men, which is in line with physiological literature. Since heart volume and muscle mass are both lower in women, less volume generates a smaller stroke volume (SV) and a higher HR is needed for the same cardiac output and oxygen supply to the body (Cardiac output = HR x SV). In contrast, maximal HR was found to be higher in men. Female's higher body fat content may explain this finding [28], as their maximal oxygen uptake normalized by body weight per heartbeat is lower than in men. A woman therefore reaches earlier maximal HR that in turn relates to lower work performance. In agreement with data from the Swiss Federal Statistical Office in 2012, a quarter of the participants presented with increased SBP [29]. Interestingly, a higher rate was revealed in males compared to females (34% vs. 11%). The Swiss Federal Statistical Office confirmed that until the age of 65 years, men tend to have higher rates of increased BP than women [29].

The present study showed that 87% of the participants met the current recommendations on physical activity of at least 30 minutes MVPA per day, clearly exceeding the findings of the Swiss Health Survey 2012 (72%) [30]. An explanation could be that in our study physical activity was measured objectively 23 hours a day, whereas the Swiss Health Survey used a self-report questionnaire, which only assessed physical activity during leisure-time.

### 4.2. Associations of Metabolic and Cardiovascular Factors with VO_2max_

This study detected a mean VO_2max_ of 40 ml/kg/min with a highly significant difference between men and women (45 ml/kg/min vs. 33 ml/kg/min). Compared to reference values for healthy nonathletes ranging from 30 to 50 ml/kg/min, the study subjects were found to be on an average level with regard to cardiorespiratory fitness [3].

Participants in the fittest group were significantly younger compared to subjects in the unfit group. A decline in VO_2max_ with age is well known with a linear 10%-decrease per decade (1% per year in men, 0.8% per year in women) after the age of 25 years [31, 32]. VO_2max_ is determined by cardiac output and arteriovenous oxygen difference, with the former probably being the driving factor for this decline. Reasons for a reduction in cardiac output with age include a notable decline in maximal HR [20] and a decrease in SV of about 10-20% compared to values at young age caused by increased peripheral resistance from reduced elasticity of the arteries and arterioles [33]. As expected, participants in the fittest group had a lower BMI and WC than those in the unfit group. This is consistent with previous findings and points to the impact of body fat on oxygen uptake and cardiorespiratory dynamics [7].

Several studies demonstrated that the likelihood of developing hypertension is lower in physically active compared to inactive individuals [34]. In contrast, this study showed a higher SBP in the fittest compared to the unfit group. A possible explanation is that mostly men were in the fittest group, who typically had a higher SBP than women. Indeed, this relationship did not substantiate when adjusted for sex. A study examining CV health in 3.000 men over a period of 16 years revealed that high HR at rest correlated with lower cardiorespiratory fitness, higher BP, higher levels of circulating blood fats, and higher body weight [35]. Our data underscore these findings, since the fittest group had a significantly lower resting HR compared to the unfit group. In addition, the fittest group showed a significantly higher increase in HR during exercise and a greater decline during recovery, indicating a substantially reduced CV risk compared to the unfit group [36]. This is further supported by the finding that the double product was lower in the fittest group. Previous studies detected a strong association between the double product and CV mortality, thereby emphasizing its prognostic value [37].

### 4.3. Regression Model to Estimate VO_2max_

VO_2max_ is a well-established measure for cardiorespiratory fitness and closely linked to all-cause mortality [10]. Despite its clinical relevance, methods for direct assessment remain time-consuming and difficult to perform in primary care [12]. This study revealed that VO_2max_ is primarily influenced by sex, WC, difference of maximal to resting HR, smoking, and age. The resulting regression model based on these readily available nonexercise variables proved accurate in estimating VO_2max_ in a healthy working population when compared to values determined by the 20-meter shuttle run test.

The present findings are in line with previous studies indicating that female sex is a strong negative predictor of VO_2max_ and associated with 10-15% lower levels than in males [38, 39]. Women not only have a smaller SV, but also a lower hemoglobin concentration [38]. Thus, less oxygen is transported per liter of SV. Another reason is that men have more mitochondria due to the higher muscle mass, which in turn results in higher oxygen metabolism [3]. However, the sex difference in this study (13 ml/kg/min) was substantially larger than in similar regression equations developed in adolescents (~5-7 ml/min/kg) [40, 41]. It could be hypothesized that differences in VO_2max_ between sexes are more pronounced in adults, when growth is completed. Our results also highlight a significant association of VO_2max_ with WC, which particularly in men has been described to be stronger than with BMI [42]. Several studies confirmed the inverse relationship of abdominal obesity with cardiorespiratory fitness [14, 42]. Furthermore, the present findings are consistent with Uth et al. [43] showing a highly significant correlation between VO_2max_ and the ratio of maximal to resting HR. This can partly be explained by exercise-induced lowering of resting HR while increasing maximal HR, which in turn results in a higher VO_2max_. By adjusting for age in the regression model, the fact that people in the fittest group were younger and would have a higher maximal HR due to their age was controlled. The negative association of VO_2max_ with smoking is again in line with previous research [5]. A trial found higher HR as well as lower VO_2max_ and anaerobic threshold in smokers, possibly due to reduced oxygen availability for the exercising muscles [44]. Moreover, age was regarded as one of the most important factors associated with VO_2max_ based on results from a maximal graded exercise test in adults aged 18 to 65 years [15]. The present results similarly point to age as a negative determinant of VO_2max_, although the effect was only 0.1 ml/kg/min decrease per year, which is less than seen in the literature (~0.3-0.5 ml/kg/min per year) [31, 32]. This discrepancy may be explained by the fact that the age effect is partly represented in the difference of maximal to resting HR, which has not been included as independent variable in other regression equations. In this study, physical activity did not appear to independently determine VO_2max_, which is supported by McMurray et al. indicating that cardiorespiratory fitness is more closely associated with the risk of CV disease than physical activity [2]. Surprisingly, neither SPB nor DBP correlated significantly with VO_2max_. This is in contrast to a prior study that showed a negative association between BP and VO_2max_ in normal weight persons [44].

### 4.4. Implications for Clinical Practice

Previous studies convincingly demonstrated that good cardiorespiratory fitness has beneficial effects on morbidity and mortality. High VO_2max_ reduces the risk of various diseases such as stroke, ischemic heart disease, hypertension, type 2 diabetes, and metabolic syndrome. Therefore, the design of a tool to estimate VO_2max_ is of pivotal importance for preventing CV and other diseases in healthy adults. Furthermore, it is evident that such tools are of little value unless they become implemented in daily clinical routine. Thus, the development of a regression model that is simple in use, solely includes readily available parameters, and does not require any specific measurement, equipment, or infrastructure is highly needed. The model derived in this study provides the potential for use in computer applications to support clinical decisions. The given examples elucidate how VO_2max_ is influenced by the individual metabolic and CV profile. Consequently, the formula contributes to further increase the accessibility to CV risk assessment and personalized advice on preventive measures.

### 4.5. Strengths and Limitations

This study included a wide range of healthy employees, providing data from a typical cross-section of the Swiss working population. Moreover, the data on mean VO_2max_ as well as VO_2max_ values stratified for sex are comparable with data from a population-based study in US employees [15].

Several limitations of the study must be taken into consideration when interpreting the results.* First*, the 20-meter shuttle run test was used to determine VO_2max_ rather than spiroergometry, which is the gold standard for direct measurement of VO_2max_ [11]. However, spiroergometry depends on trained staff and is labor-intensive and time-consuming and therefore not feasible for evaluating large populations [12]. A recent study verified that the 20-meter shuttle run test was accurate in predicting VO_2max_ in healthy adults, as the results showed a strong and highly significant correlation between the number of achieved shuttles and directly measured VO_2max_ [45].* Second*, the regression model for estimating VO_2max_ developed in this study may have limited generalizability, given that it was based on healthy younger adults in Switzerland. Thus, future research should focus on other populations in order to refine the model, e.g., elderly or diseased people. Nevertheless, the different age groups included here accurately reflect the distribution of the Swiss working population, where preventive measures may be most effective for maintaining health and work productivity.* Third*, it remains uncertain if individuals with great interest in health issues and fitness were more willing to participate in the study than others, thereby resulting in a possible selection bias. A lack of motivation among the low fit individuals may also have influenced the results, as they might have terminated the 20-meter shuttle run test earlier resulting in lower maximal HR and VO_2max_.* Fourth*, only one BP measurement was performed at rest and during recovery, respectively, whereas the 2013 ESH guidelines suggest at least two measurements [22]. This may have led to a lack of relationship between VO_2max_ and BP.* Fifth*, the present study was cross-sectional in nature, which provided a snapshot of the relation between cardiometabolic factors and VO_2max_. However, cause-effect relationships are difficult to ascertain. To overcome this limitation in future, longitudinal studies are required.

## 5. Conclusions

This study introduced a simple model for estimating VO_2max_ based on nonexercise parameters without needing a time-consuming, cost-intense, and physically demanding direct assessment of VO_2max_. Thus, the study enabled a new way to efficiently and effectively evaluate VO_2max_ as part of daily clinical routine. However, determining an individual's VO_2max_ is not only intended to estimate cardiorespiratory fitness, but also to identify future health risks associated with a low VO_2max_.

## Figures and Tables

**Table 1:** Characteristics of study participants.

Variables	Total (n = 303) Mean (± SD) or n (%)	Male (n = 190) Mean (± SD) or n (%)	Female (n = 113) Mean (± SD) or n (%)
*Age [yrs]*	33 (12)	33 (13)	35 (12)
*Current smokers*	64 (21%)	46 (24%)	18 (16%)
*BMI [kg*/*m*^2^*]*	24 (3)	25 (3.2)	23 (4)
Normal	187 (62%)	106 (56%)	81 (72%)
Overweight [≥25 kg/m^2^]	95 (32%)	67 (35%)	28 (25%)
Obesity [≥30 kg/m^2^]	21 (7%)	17 (9%)	4 (4%)
*WC [cm]*	86 (11)	89 (10)	80 (11)
Normal	200 (66%)	139 (74%)	61 (53%)
Abdominal obesity [m >94 cm, f >80 cm]	102 (34%)	50 (27%)	52 (46%)
*Heart rate [bpm]*			
HR_rest_ [bpm]	72 (13)	70 (13)	74 (13)
HR_max_ [bpm]	183 (15)	187 (14)	178 (15)
HR_recovery_ [bpm]	106 (15)	108 (14)	102 (16)
*Blood pressure [mmHg]*			
SBP_rest_ [mmHg]	136 (15)	140 (12)	128 (18)
SBP_recov_ [mmHg]	164 (19)	171 (17)	152 (16)
DBP_rest_ [mmHg]	82 (10)	82 (9)	80 (10)
DBP_recov_ [mmHg]	84 (10)	84 (10)	83 (9)
Normal BP [<140/90 mmHg]	173 (57%)	86 (45%)	87 (77%)
Increased SBP [≥140 mmHg]	76 (25%)	64 (34%)	12 (11%)
Increased DBP [≥90 mmHg]	9 (3%)	8 (4%)	1 (1%)
Increased SBP and DBP [≥140/90 mmHg]	45 (15%)	32 (17%)	13 (12%)
BP lowering drug therapy	10 (3%)	7 (4%)	3 (3%)
*VO*_2*max*_ *[ml/kg/min]*	40 (10)	45 (8)	33 (7)
<P25	71 (23%)	13 (7%)	58 (51%)
P25-75	155 (51%)	10 (55%)	50 (44%)
>P75	77 (25%)	72 (38%)	5 (4%)
*Daily MVPA [min*/*d]*	121 (96)	144 (106)	83 (61)
Insufficient [<30 min/d]	37 (13%)	16 (9%)	21 (20%)
Sufficient [≥30 min/d]	242 (87%)	157 (91%)	85 (80%)

BMI, body mass index; BP, blood pressure; BPM, beats per minute; DBP, diastolic blood pressure; HR, heart rate; MVPA, moderate-to-vigorous physical activity; <P25, unfit group; P25-75, middle group; >P75, fittest group; SBP, systolic blood pressure; SD, standard deviation; VO_2max_, maximal oxygen uptake; WC, waist circumference.

**Table 2:** Forward-stepwise multiple linear regression analysis with VO_2max_ as dependent variable.

Model: Adjusted R^2^ = 0.647	B	SE B	ß	p-value
*Constant*	*77.947*	*5.322*		*<0.001*
Sex [male vs. female]	-13.374	0.852	-0.699	<0.001
WC	-0.338	0.037	-0.403	<0.001
HR_max_-to-HR_rest_	0.121	0.022	0.234	<0.001
Smoking status [never vs. yes]	-3.938	0.857	-0.171	<0.001
Age [yrs]	-0.098	0.032	-0.131	0.002

B, unstandardized regression coefficient; ß, standardized beta coefficient; HR_max_-to-HR_rest_, difference of maximal to resting heart rate; SE, standard error; VO_2max_, maximal oxygen uptake; WC, waist circumference.

Excluded variables: body mass index, systolic and diastolic blood pressure, double product, difference of maximal to recovery heart rate, moderate-to-vigorous physical activity, and daily working hours.

## Data Availability

The data used to support the findings of this study are available from the corresponding author upon request.
